# Accuracy Assessment of Planned Versus Actual Trajectories in Robotic-Assisted Spine Surgery Utilizing Perioperative O-Arm CT Scans

**DOI:** 10.7759/cureus.107146

**Published:** 2026-04-16

**Authors:** Mirant B Dave, Mikeson Panthackel, Bharatkumar R Dave, Ajay Krishnan, Shivanand C Mayi, Ravi Ranjan Rai, Arjit Vashishtha, Amritesh Singh, Saurabh S Kulkarni, Yogenkumar Adodariya

**Affiliations:** 1 Spine Surgery, Stavya Spine Hospital and Research Institute, Ahmedabad, IND; 2 Spine, Bhavnagar Institute of Medical Sciences (BIMS), Bhavnagar, IND; 3 Orthopaedics, University College of Medical Sciences and Guru Teg Bahadur Hospital, Delhi, IND; 4 Orthopaedics, Mahatma Gandhi Medical College and Research Institute, Aurangabad, IND

**Keywords:** accuracy, mazor x, minimally invasive spine surgery, pedicle screw, robotics

## Abstract

Study design

A prospective cohort study.

Objectives

To find the deviations in entry point, axial angulation, and sagittal angulation between the planned trajectories versus the achieved trajectories in Mazor-X robotic spine surgery.

Methods

This study was conducted from December 2023 to June 2025, including 250 patients undergoing robotic-assisted pedicle screw fixation. Planned and actual screw trajectories were compared using perioperative O-arm CT scans.

Results

A total of 1170 screws were inserted (96 thoracic, 1074 lumbar). Mean entry deviation was 2.27 ± 1.5 mm, axial angular deviation 3.7° ± 3.7°, and sagittal deviation 3.4° ± 3.2°. A clinically acceptable placement rate of 99.7% was achieved, with only four breaches requiring revision. Operative time and screw insertion time decreased with experience, reflecting a short learning curve.

Conclusion

Robotic guidance using the Mazor X Stealth Edition (Medtronic, 2018, Memphis, Tennessee) ensures highly accurate and safe pedicle screw placement in spine surgery.

## Introduction

Spine surgery is a delicate procedure in which precision is paramount. Small inaccuracies can lead to devastating complications for patients. The use of newer technologies such as computer-assisted navigation and robotic-assisted surgery has significantly reduced the risk of complications [1].

Since the introduction of the first robot in spine surgery, the Mazor SpineAssist (Mazor Robotics Ltd., Caesarea, Israel) in 2004, the role of robotics in spine surgery has been steadily growing [2]. Robotic systems offer several benefits, including increased accuracy (98% accurate), elimination of hand tremors, reduced surgeon fatigue, and smaller incision sizes [3]. Cohort studies and meta-analyses have shown that robotic-assisted percutaneous pedicle screw placement results in lower blood loss, reduced radiation exposure to the surgeon and OT personnel, and improved accuracy compared to screws inserted via the Wiltse or open approach [4,5].

Though a similarly high level of accuracy for pedicle screw insertion can be achieved using 3D navigation alone (95%), the precision of robotic-assisted screws is significantly higher than that of navigation-guided screws (85% vs 65% grade A screws) [6].

Studies assessing the accuracy and deviation of planned trajectories in robotic spine surgery have mostly been conducted using previous generations of Mazor robots (Renaissance, Mazor X, Mazor Robotics Ltd., Caesarea, Israel) and ExcelsiusGPS (Globus Medical, Inc., Audubon, Pennsylvania). These previous studies show high accuracy for robotic-assisted pedicle screws (approximately 2 mm). However, studies on the newest version robot, the Mazor X Stealth Edition (Medtronic, 2018, Memphis, Tennessee) (with navigation), are currently limited [7].

The present study aims to determine the level of accuracy in executing planned trajectories using the Mazor X Stealth Edition robot (Medtronic, 2018, Memphis, Tennessee) by comparing them with the actual trajectories achieved during the procedure.

## Materials and methods

This study was done at Stavya Spine Hospital and Research Institute, Gujarat. Approval was taken from the institutional ethics committee, and informed consent was taken from all patients participating in the study. In this prospective cohort study, data were collected from all patients undergoing robotic spine surgery who consented to participate in the study at a tertiary spine care institute from December 2023 to June 2025. Exclusion criteria included scoliosis with a Cobb angle of >20°. All patients underwent robot-assisted spine surgery using the Mazor X Stealth Edition (Medtronic, 2018, Memphis, Tennessee). All the patients underwent surgery by a single trained spine surgeon. Demographic and clinical information, including age, sex, diagnosis, and comorbidities, was collected. Intraoperative data were collected in real-time during robotic spine surgeries. This study comprises the first 250 cases of robotic-assisted spine surgery at our institution.

Surgical technique

At our institution, first, the robot is docked onto the operating table with the arm pointing parallel to the table, allowing for space to position the patient. Following this, the patient is positioned in the prone position, ensuring that the surgical site is within the reach of the robotic arm. After painting and draping the patient, the robot is also draped. Following the decompression part of the procedure, the O-arm is brought in for intraoperative CT acquisition. The scan is then loaded onto the Mazor X station, and the planning of screw trajectories is carried out. After planning, drilling, and tapping are done, the screws are placed using guidewires. After the insertion of all screws and confirmation of their positions on the post-instrumentation O-arm CT (second spin), the robot is unmounted. The wound is closed in layers.

Trajectory analysis

**Figure 1:**
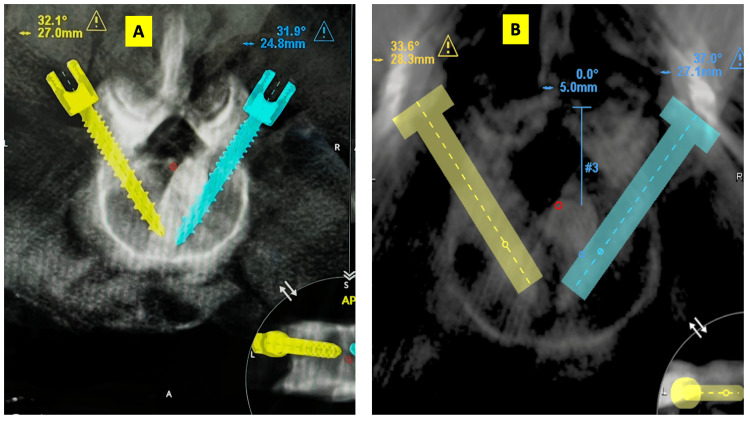
Image showing pre-operative planning and post-operative parameter acquisition from CT scans uploaded into the Mazor X software. (1A) Planned trajectory coordinates noted from the robot console. The yellow and blue screws represent the planned right and left screw trajectories, respectively. (1B) Post-operative CT assessment. Yellow and blue virtual cylinders are placeholders used to measure the angles within the software. These templates are superimposed over the visible screws in the post-operative scan so the final parameters can be recorded.

Planned trajectory coordinates and actual trajectory coordinates were recorded and compared to assess screw accuracy. Any breaches were graded according to the Gertzbein-Robbins classification [8]. The parameters of angulation and distance from the midline in axial images and sagittal angulation were collected from the planning software. Post-operative CT datasets were imported into the Mazor X planning workstation. For each pedicle screw, the image slice demonstrating the maximal longitudinal profile was selected for assessment. A virtual screw template was manually superimposed onto the post-operative image; alignment was standardized by matching the virtual screw head with the tulip of the inserted screw and overlaying the central axis guide along the shaft of the implanted screw. The resulting coordinates were recorded and compared against the pre-operative planned values. An example of such comparison is shown in Figure 1. The deviations from the planned trajectories were noted, and the averages were calculated. Angular deviations in both axial and sagittal orientations were also recorded. To minimize observer bias, measurements were performed independently by two observers who were blinded to each other’s measurements and to the planned trajectory data at the time of assessment. Each observer recorded measurements separately without mutual consultation.

Entry-Point Deviation (mm): Defined as the linear difference in the distance from the anatomical midline to the screw entry point when comparing the CT-based intraoperative planning trajectory vs achieved trajectory. Axial Angular Deviation (°): Defined as the absolute difference in the trajectory angle between the planned and inserted screw within the axial plane. Sagittal Angular Deviation (°): Defined as the absolute difference in the trajectory angle between the planned and inserted screw within the sagittal plane.

Inter-observer reliability assessment

Inter-observer reliability for trajectory deviation measurements was assessed using the intraclass correlation coefficient (ICC). A two-way random-effects model with absolute agreement for single measurements (ICC: 2,1) was used, as both observers evaluated all screws and were considered representative of a larger population of potential raters. ICC analysis was performed separately for entry-point deviation, axial angular deviation, and sagittal angular deviation. Reliability was interpreted according to established criteria, with ICC values <0.50 considered poor, 0.50-0.75 moderate, 0.75-0.90 good, and >0.90 indicative of excellent agreement.

Statistical handling of deviation measurements

Deviation measurements were analyzed at the screw level. Continuous variables were expressed as mean ± standard deviation. Inter-observer reliability was reported with corresponding 95% confidence intervals. Statistical analyses were performed using IBM SPSS Statistics for Windows, Version 20.0 (Released 2011; IBM Corp., Armonk, New York), and statistical significance was defined as p < 0.05. Statistical analyses to quantify the accuracy of trajectory execution based on the data collected during the study were performed.

Power analysis: Sample size calculation was based on estimating the mean entry point deviation with a precision of 0.2 mm and a 95% confidence interval. Assuming a standard deviation of 1.5 mm based on prior robotic literature [9], a minimum of 216 screws was required.

## Results

A total of 255 patients initially underwent planned robotic-assisted spine surgery, of whom five patients required intraoperative conversion to three-dimensional navigation due to robotic abandonment. Consequently, 250 patients were included in the final analysis conducted between December 2023 and June 2025. A total of 1170 screws were placed, of which 96 were thoracic screws and 1074 were lumbar screws.

The mean age of the study population was 58.03 years. Of the total patients, 90 were male, and 160 were female. The median body mass index (BMI) was 27.9. Characteristics of the study group are summarized in Table 1.

**Table 1:** Characteristics of the study population. Values given in n (%) format.

Parameter	Variable	Value
Age (years) mean ± SD		58.03 ± 9.0
Sex (M/F)	Male	90 (36%)
	Female	160 (64%)
BMI mean ± SD		27.9± 6.5
Pathology	Listhesis	83 (34%)
	Recurrent PID/PID with instability	8 (3%)
	Stenosis requiring wide decompression (iatrogenic instability)	74 (30%)
	Stenosis+ Listhesis	82 (32%)
	Multilevel	3 (1%)
Number of levels fused	1 level	205 (82%)
	2 levels	45 (18%)

The average duration of surgery was 111.07 minutes. In the first five surgeries, the average time for surgery was 108 minutes, which reduced to 94 minutes in the last five surgeries. To ensure a standardized comparison of operative times, both the initial five and final five cases were single-level MIS TLIF procedures requiring the placement of four pedicle screws each. The mean time for inserting each screw decreased from 13 minutes in the initial surgeries to an average of 5 minutes per screw from the 20th surgery onward. The mean blood loss was 121 mL. Only four screws in total had to be revised due to breaches.

The mean hospital stay was 3.59 days. The trajectory variation of the screws was assessed, and only minimal discrepancies were found. Entry point deviation averaged 2.27 ± 1.5 mm. Axial angular deviation was 3.7° ± 3.7°, and sagittal angular deviation was 3.4° ± 3.2°.

Inter-observer reliability for trajectory deviation measurements was assessed using the ICC using a two-way random-effects model with absolute agreement for single measurements (ICC: 2,1). The ICC for entry-point deviation was 0.91 (95% CI: 0.88-0.94) and for axial angular deviation was 0.90 (95% CI: 0.87-0.93), indicating excellent inter-observer agreement. The ICC for sagittal angular deviation was 0.82 (95% CI: 0.77-0.86), reflecting good agreement between observers. The results of the interobserver analysis are shown in Table 2.

**Table 2:** Inter-observer reliability for trajectory deviation measurements. Two observers evaluated 1170 screws, and the inter-observer reliability of trajectory deviation measurements was assessed using the ICC based on a two-way random-effects model with absolute agreement for single measurements (ICC: 2,1). Confidence intervals represent 95% limits. Statistical significance was defined as p < 0.05. ICC: intraclass correlation coefficient.

Deviation parameters	ICC (2,1)	95% confidence interval
Entry-point deviation (mm)	0.91	0.88–0.94
Axial angular deviation (°)	0.90	0.87–0.93
sagittal angular deviation (°)	0.82	0.77–0.86

The breaches were graded according to the Gertzbein-Robbins classification [8]. Grade B breaches were noted in one case, with all screws (four screws) breaching inferiorly. These were redirected under navigation guidance. Excluding these four screws, the remaining 1166 out of 1170 screws were classified as Gertzbein A or B, giving us a clinically acceptable screw rate of 99.7%. The number of screws in each grade are noted in Table 3.

**Table 3:** Distribution of pedicle screws according to the Gertzbein-Robbins classification. This table summarizes the distribution of pedicle screws across Gertzbein-Robbins grades. All values are expressed as N (%) of screws in each grade.

Grade	Number of screws
Grade A (no breach)	990 (84.7%)
Grade B (<2 mm breach)	176 (15%)
Grade C (<4 mm breach)	3 (0.2%)
Grade D (<6 mm breach)	1 (0.1%)
Total number of screws	1170 (100%)

## Discussion

Although robotic surgery has long been utilized in other specialties such as gynecology, urology, and general surgery, the adoption of this technology in spine surgery has been relatively late and slow. The first robot, the Mazor SpineAssist (Mazor Robotics Ltd., Caesarea, Israel), was introduced in 2004. Multiple iterations of the robot from Mazor Robotics Ltd. have followed, with the most recent being the Mazor X Stealth Edition (Medtronic, 2018, Memphis, Tennessee) [3].

Analyzing 1170 screws in 250 patients, this series constitutes one of the largest datasets evaluating trajectory deviation in the Mazor X platform to date. Temporal analysis demonstrated a marked reduction in operative duration, with the mean screw insertion time decreasing from 13 minutes in the initial cohort to 5 minutes in the final cases. The time required for each screw began to stabilize from the 15th surgery onward, with occasional fluctuations. The average duration of surgery was reduced from 108 minutes in the initial five cases to 94 minutes per case in the last five cases. This reduction in surgical time can be attributed to the learning curve associated with the adoption of the new procedure. One study on the learning curve focused on the successful placement of pedicle screws and found that a 90% success rate for robotic pedicle screw insertion was achieved by the 30th case, suggesting the learning curve to be approximately 30 cases [10].

The angular and distance deviations between the planned trajectories and the post-operative CT scans were minimal, with averages of 2.27 mm and 3.7°, respectively. One comparable study has reported deviations of 2.0 mm in entry point and 2.2° in axial angulation; however, that study was conducted using an older version of the Mazor SpineAssist (Mazor Robotics Ltd., Caesarea, Israel) robot [9]. Studies on the ExcelsiusGPS robot (Globus Medical, Inc., Audubon, Pennsylvania) have shown a total deviation of 3.2 mm and an angular deviation of 2.4° [7].

Inter-observer reliability analysis demonstrated excellent agreement for entry-point deviation and axial angular deviation measurements, while sagittal angular deviation showed good agreement. The comparatively lower reliability observed for sagittal angular measurements may reflect increased sensitivity to slice selection, metal artifact, and minor differences in defining the screw axis in the sagittal plane on post-operative CT imaging. Nevertheless, overall reliability findings support the reproducibility of the predefined measurement protocol for assessing planned versus actual pedicle screw trajectory deviations.

In our study, the rate of clinically acceptable screws was 99.6%. Similar high accuracies have been observed in multiple recent studies using robotic guidance [9,11-14]. Almost all reported accuracy rates were between 98% and 99%. One study, in particular, had a lower accuracy rate of 91%, possibly due to the use of a bed-mounted frame [15]. Extensive comparative literature, including a comprehensive meta-analysis by Fan et al., consistently demonstrates that robotic-assisted pedicle screw placement offers significantly higher accuracy than conventional freehand techniques. Comparative studies between robotic and freehand screw placement have demonstrated a clear advantage in favor of robotic systems [12,16]. The accuracy rates of screw placement have steadily increased with advancements in the precision, stability, and maneuverability of robotic arms. Studies comparing the Mazor X and Renaissance platforms showed better accuracy with the Mazor X, especially for complex trajectories such as S2AI screws (1.2% breach rate with Mazor X vs. 9.5% with Renaissance) [17].

We had only four screws with significant inferior breaches, which were later repositioned. The remaining screws had acceptable trajectories. Vidyadhara et al. have stated that there is a significant risk of screw inaccuracies in cases of inadvertent patient movement or reference frame displacement [14].

We had five cases (2%) of robotic abandonment. The most common cause was the inability to achieve the planned trajectory using the robotic arm. Similar studies on robotic abandonment have been reported in the literature. In a study by Keric et al., failed registration was the primary cause. Obesity and osteopenia were identified as possible contributors to failed registration. In a study by Hu and Lieberman, robotic assistance was aborted in 110 out of 949 patients, either due to registration failure or trajectory errors caused by skiving [10]. The rates of abandonment have significantly decreased with newer systems, from 22.7% on the Renaissance platform to 2.3% in the Mazor X system [17].

The availability of 3D visualization in the Mazor X Stealth Edition (Medtronic, 2018, Memphis, Tennessee), a navigated robotic system, further reduces fluoroscopy time (Stealth: 7.2 sec vs X: 10.4 sec) and abandonment rates (Stealth: 0% vs X: 2.2%) when compared with the Mazor X non-navigated robotic system. This can be attributed to a reduced need for double-checking screw trajectories using fluoroscopy in non-navigated systems [18].

The limitations of our study are first; this is a single-center study. Second, the use of Mazor X software to manually overlay the trajectories is subject to observer variability due to artifacts in post-operative CT scans, making it difficult to accurately identify the trajectories and increasing the potential for human error.

## Conclusions

This study confirms that the Mazor X Stealth Edition (Medtronic, 2018, Memphis, Tennessee) system provides high-precision instrumentation, evidenced by an excellent accuracy rate and minimal trajectory deviations. The operative data also highlight a short learning curve.
